# Effects of Secondary Metabolite Extract from *Phomopsis occulta* on β-Amyloid Aggregation

**DOI:** 10.1371/journal.pone.0109438

**Published:** 2014-10-02

**Authors:** Haiqiang Wu, Fang Zhang, Neil Williamson, Jie Jian, Liao Zhang, Zeqiu Liang, Jinyu Wang, Linkun An, Alan Tunnacliffe, Yizhi Zheng

**Affiliations:** 1 College of Life Sciences, Shenzhen University, Shenzhen, China; 2 Department of Chemical Engineering and Biotechnology, University of Cambridge, Cambridge, United Kingdom; 3 College of Pharmacy, Guilin Medical University, Guilin, China; 4 School of Pharmaceutical Science, Sun Yat-sen University, Guangzhou, China; University of S. Florida College of Medicine, United States of America

## Abstract

Inhibition of β-amyloid (Aβ) aggregation is an attractive therapeutic and preventive strategy for the discovery of disease-modifying agents in Alzheimer's disease (AD). *Phomopsis occulta* is a new, salt-tolerant fungus isolated from mangrove *Pongamia pinnata* (L.) Pierre. We report here the inhibitory effects of secondary metabolites from *Ph. occulta* on the aggregation of Aβ42. It was found that mycelia extracts (MEs) from *Ph. occulta* cultured with 0, 2, and 3 M NaCl exhibited inhibitory activity in an *E. coli* model of Aβ aggregation. A water-soluble fraction, ME0-W-F1, composed of mainly small peptides, was able to reduce aggregation of an Aβ42-EGFP fusion protein and an early onset familial mutation Aβ42E22G-mCherry fusion protein in transfected HEK293 cells. ME0-W-F1 also antagonized the cytotoxicity of Aβ42 in the neural cell line SH-SY5Y in dose-dependent manner. Moreover, SDS-PAGE and FT-IR analysis confirmed an inhibitory effect of ME0-W-F1 on the aggregation of Aβ42 *in vitro*. ME0-W-F1 blocked the conformational transition of Aβ42 from α-helix/random coil to β-sheet, and thereby inhibited formation of Aβ42 tetramers and high molecular weight oligomers. ME0-W-F1 and other water-soluble secondary metabolites from *Ph. occulta* therefore represent new candidate natural products against aggregation of Aβ42, and illustrate the potential of salt tolerant fungi from mangrove as resources for the treatment of AD and other diseases.

## Introduction

Alzheimer's disease (AD) is a devastating condition leading to progressive cognitive decline, functional impairment and loss of independence, and is the major cause of dementia in the elderly worldwide [1]. Its prevalence will continue to increase as life expectancy increases. AD therefore represents a major and rising public health concern. However, as none of the medicines currently in use are able to cure this neurodegenerative disorder [2], understanding its etiology and developing new protective medicines have become the primary research goals in AD research.

Many clinicopathological studies have demonstrated that the deposition of beta-amyloid (Aβ) peptides, fragments of the amyloid precursor protein (APP), in brain parenchyma and cerebral blood vessels is one of the hallmarks of AD [3], [4]. Although the molecular mechanism of its involvement in the development and progression of AD is not clear, a critical role for Aβ is universally acknowledged [5]. Aβ fibrils were once thought to be the main molecular culprit in AD, but recent studies show a more decisive correlation between the levels of soluble, non-fibrillar Aβ oligomers and the extent of synaptic loss and cognitive impairment [6]–[8]. Compared with Aβ fibrils and plaques, Aβ oligomers are more potent as neurotoxins that cause disruption of neuronal synaptic plasticity [9], [10]. The relationships between Aβ peptides, oligomerisation, cellular dysfunction and AD suggest that inhibition of Aβ oligomerisation might lead to novel therapeutics for the treatment of AD [11].

In addition to chemical pharmacological agents, bioactive extracts derived from natural products are attracting increasing attention in the search for new effective agents for the treatment of AD. Examples of such extracts that, when administered, led to inhibition of Aβ aggregation and related downstream pathological responses include aged garlic extract (AGE) [12], *Ginkgo biloba* extract (EGb761) [13], fungal endophytic extracts of Malaysian medicinal plants [14], *Alpinia galanga* (L.) fractions [15], Yokukansan extract [16], coffee extract [17], Samjunghwan extract [18], *Paeonia suffruticosa* extract [19], GEPT (a combination of extracts of ginseng, *Epimedium*, *Polygala* and tubers of the *Curcuma* genus) [20].

Marine microorganisms are a source of potentially useful natural extracts for the treatment of multifaceted diseases such as AD [21], [22], and we focus here on microbes associated with mangroves, which are salt-tolerant, woody trees that grow in coastal habitats. Recently, we isolated and identified a new salt-tolerant endophytic fungus, *Phomopsis occulta SN3-2* (CCTCC No. 2011044), from mangrove *Pongamia pinnata* (L.) Pierre, and have assessed water-soluble secondary metabolites from *Ph. occulta* for inhibitory effects on the aggregation of Aβ42 in mammalian cells and *in vitro*. Here we show that a bioactive fraction, ME0-W-F1, from *Ph. occulta* mycelia extract can reduce formation of high molecular weight (HMW) Aβ42 oligomer and tetramer *in vitro* by inhibiting the formation of β-sheet secondary structure. Moreover, ME0-W-F1 is able to reduce the neurotoxic effect of Aβ42 in SH-SY5Y cells.

## Materials and Methods

### Reagents

*Phomopsis occulta* SN3-2 is a new species of fungus, identified tentatively by the Institute of Microbiology, Chinese Academy of Sciences, and maintained at the Shenzhen Key Laboratory of Microbial & Genetic Engineering, Shenzhen University, Shenzhen, China and also at the China Center for Type Culture Collection (CCTCC No. 2011044). Synthetic Aβ42 peptide was purchased from GenScript USA Inc. (Piscataway NJ, USA). (−)-Epigallocatechin gallate (EGCG) was obtained from Sigma-Aldrich Company Ltd.; stock solutions (10 mM) were freshly prepared in water. Diaion-20 resin hexafluoro-2-propanol (HFIP; Sigma) and all other chemicals were of reagent grade and commercially available.

### Culture of *Phomopsis occulta* and preparation of its secondary metabolite extracts

Axenic cultures of *Ph. occulta* were maintained on potato dextrose agar. The cultures were transferred to liquid medium LB for 5–7 days, and then incubated in LB medium containing 0, 1, 2 or 3 M NaCl at 28°C without shaking for 40 days. These cultures were separated by filtration into mycelia and filtrates. The filtrates were concentrated to 2 L below 45°C in the dark, and extracted five times by shaking with an equal volume of ethyl acetate (EtOAc). After drying using anhydrous Na_2_SO_4_, collection and evaporation of EtOAc at 50°C *in vacuo* using a rotary evaporator (RV06-ML 1-B, IKA, Germany) yielded the fermentation broth extracts BE0, BE1, BE2 and BE3 (corresponding to cultures at 0, 1, 2 or 3 M NaCl, respectively). The mycelia were dried under vacuum and extracted three times using 2 L methanol for 72 h. Combination and evaporation of methanol yielded the mycelia extracts ME0,ME1, ME2 and ME3 (corresponding to cultures at 0, 1, 2 or 3 M NaCl, respectively).

### *Escherichia coli* cell model

*E. coli* cell models of Aβ aggregation have been developed by others previously [23]–[25]. Briefly, *E. coli* cultures capable of producing a secretable form of Aβ42 fused to β-lactamase were grown overnight in LB supplemented with chloramphenicol (Cam) and then diluted 1∶100 and grown for another 3 h at 37°C. These exponential phase cultures were diluted 1∶50 in 96-well plates containing LB supplemented with 12.5 µg/mL Cam, 1 mM isopropyl-β-D-thiogalactopyranoside, 50 µg/mL ampicillin (Amp) and, as required, 200 µg/mL test samples, and EGCG was used as positive control (100 µg/ml). The plates were incubated at 37°C for 20 h without shaking. The OD_600_ was read and relative growth rate (%) calculated according to the following formula.
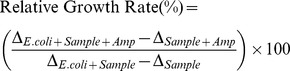



Here, Δ_E.coli+Sample+Amp_ and Δ_E.coli+sample_ represent the changes in OD_600_ in *E. coli* cell and sample interaction systems after 20 h in the presence of Amp or not, respectively. Δ_Sample+Amp_ and Δ_Sample_ represent the changes in OD_600_ in sample systems after 20 h in the presence of Amp or not, respectively. *E. coli* cells are normally killed by Amp because they are unable to export β-lactamase linked to aggregated Aβ42 peptide. If Aβ42 aggregation is inhibited, β-lactamase can be exported and degrade Amp, allowing cell growth.

### Purification of active fractions and identification by TLC

For the active *Ph. occulta* secondary metabolite extract, extraction and column chromatography were used for further purification. The active fraction ME0 was distributed between n-butyl alcohol and water phases. The water soluble components, ME0-W, were separated by column chromatography filled with Diaion-20 resin. Methanol/water was used as mobile phase, and five fractions (ME0-W-F1 to F5; 0, 5, 10, 30 and 50% methanol/water respectively (v/v)) were collected when the gradient elution was finished. Components soluble in n-butyl alcohol were not separated because of the absence of bioactivity. The inhibitory effect of these fractions on Aβ42 aggregation was assessed using the *E. coli* model described above.

The bioactive fraction, ME0-W-F1 (10 µl), was applied to cellulose precoated (20×20 cm) thin layer chromatography (TLC) plates (Merck, Germany). TLC plates were developed in a chloroform∶methanol∶water system (1∶3∶1 v/v), then air dried and visualized with iodine. Dried TLC plates were sprayed with ninhydrin reagent and heated at 80°C for 6 min. Peptide complexes became visible as intensely pink and purple-coloured bands and spots [26].

### Cell toxicity studies

SH-SY5Y cells were maintained in Ham's F12 and DMEM medium, mixed in a 1∶1 ratio, containing 2 mM glutamine, 1% nonessential amino acids, 500 µg/mL penicillin/streptomycin and 15% FBS, in an atmosphere of 5% CO_2_. Cells were transferred to a sterile 96-well plate with approximately 25000 cells per well and allowed to acclimatize for 48 h. The Ham's F12/DMEM medium was removed by suction and replaced with Optimem medium (100 µL/well) containing either no ME0-W-F1 or ME0-W-F1 (10, 100 and 200 µg/mL, in phosphate-buffered saline (PBS): 137 mM NaCl, 2.7 mM KCl, 6.5 mM Na_2_HPO_4_, 1.76 mM KH_2_PO4, pH 7.4). The cells were left for 24 h and then assessed using the MTT assay. For the protective effects of ME0-W-F1 on SH-SY5Y cells against Aβ42 aggregation, the Ham's F12/DMEM medium was replaced with Optimem medium (100 µL/well) containing either no Aβ or Aβ42 (10 µM), with and without the ME0-W-F1 (10, 100 and 200 µg/mL).

### Flp-In T-REx 293 anti-aggregation assay

The Flp-In T-REx 293 (Invitrogen) cell line, a derivative of HEK293 cells containing a stably integrated FRT site and a TetR repressor, was maintained in DMEM media (Sigma D6171) supplemented with 10% fetal bovine serum (FBS), 5 mM L-glutamine, 5 µg/ml blasticidin. T-REx 293 cells were grown at 37°C under a 5% CO_2_ atmosphere. The anti-aggregation screen was performed essentially as described [27]. Briefly 20,000 cells per well were seeded into a 24-well plate and allowed to attach for 48 h. Transient transfections were performed using GeneJammer (Agilent Technologies) as per manufacturer's instructions with either 0.75 µg of pcDNA3-Aβ42–EGFP [27] or pATNRW20. The latter construct expresses an early onset familial form of Aβ42 (Aβ42E22G) fused with the fluorescent protein mCherry (pcDNA3.3-Aβ42E22G-mCherry, N. Williamson et al., in preparation). ME0-W-F1 was added three hours post-transfection at the indicated concentrations, and gene expression was allowed to proceed for a further 48 h. An equivalent volume of dimethyl sulfoxide (DMSO) was used as a negative control and 10 µM epigallocatechin gallate (EGCG), a compound known to inhibit amyloid formation, was used as a positive control.

Quantification of Aβ42 aggregates was performed as described previously [27]. Approximately 200 GFP (Aβ42) or mCherry (Aβ42E22G) positive cells were counted for each treatment and cells were scored as positive if they contained one or more aggregates. Images were acquired on an Olympus IX81 inverted wide field microscope and all experiments were performed in triplicate and odds ratio analysis of aggregation data was performed using the statistical package GraphPad Instat 3. The nature of Aβ42 aggregates was also demonstrated by confocal microscopy, performed as described [27].

### SDS-PAGE analysis

Preparation of synthetic Aβ42 solution was carried out according as described [28], [29]. Briefly, Aβ42 peptides were dissolved in hexafluoro-2-propanol (HFIP) for 10–12 h with shaking, sonicated for 15 min, lyophilized, and redissolved in DMSO. Aβ42 concentrations were determined by OD_280_ in a Nanodrop 8000 spectrophotometer (Thermo Fisher) after diluting with PBS (pH 7.4).

The inhibitory effect of ME0-W-F1 on Aβ42 fibril formation was monitored by sodium dodecyl sulfate polyacrylamide gel electrophoresis (SDS-PAGE) under reducing conditions on 15% Tricine gels (Invitrogen) followed by Coomassie blue staining. In each experiment, Aβ42 solution was incubated with ME0-W-F1 at 37°C and 8 µl samples were removed at various time points, then pooled and analyzed by SDS-PAGE. Gel band intensities were quantified using Quantitative One software (Bio-Rad).

### Fourier transform infrared (FTIR) spectroscopy

Measurements and evaluation were as described [25]. Spectra were collected on a NICOLET-6700 (Thermo Nicolet, USA) spectrometer at room temperature using a CaF_2_ cell with a 50 µm Teflon spacer. Aβ42 stock solution (10 mg/mL in DMSO) was prepared according to section 2.7. ME0-W-F1 was prepared in DMSO at a concentration of 1 mg/mL. Mixtures were prepared by addition of Aβ42 and ME0-W-F1 stock solutions to unbuffered D_2_O in a mass ratio of 1∶1 and measurements taken at various time points. IR spectra were collected at 2 cm^−1^ resolution. Electrode readings were uncorrected for deuterium effects. CO_2_ was removed and the air moisture inside the chamber was reduced by flushing the chamber with nitrogen gas. During each experiment, spectra were scanned 32 times over the range 4000–400 cm^−1^. In some cases, the residual overlapping band was eliminated by subtraction from the final spectrum. The OMNIC software package (Thermo Nicolet, USA) was used for analysis of FT-IR spectra. Second derivative spectra were generated by using a 9-data point (9 cm^−1^) function included in the OMNIC software package.

### Data analysis

The data were expressed as mean ± SD, or mean of means ± SE, and were evaluated by two-way analysis of variance (ANOVA) followed by a post hoc test, or *t*-test. P<0.05 was considered to be significant.

## Results

### Preparation of *Ph. occulta* secondary metabolite extracts and screening of bioactive fractions

**Figure 1:**
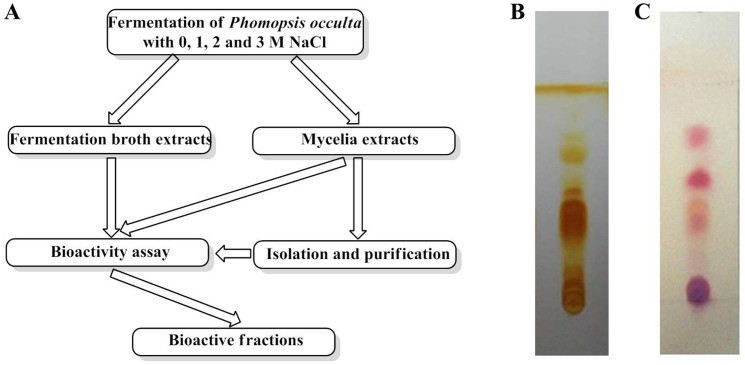
Preparation and analysis of secondary metabolites produced by *Phomopsis occulta*. A: Proposed strategy for preparation and screening of bioactive fractions from secondary metabolite extracts. B & C: Identification of ME0-W-F1 components by TLC analysis. B: visualized by iodide. C: visualized by ninhydrin.

*Ph. occulta* is a salt-tolerant fungus and we established LB cultures at various concentrations of NaCl, i.e. 0, 1, 2 or 3 M. However, fermentation was affected by salt concentration, with growth rate in the order: 1>0>2>3 M NaCl. After filtration, fermentation broth extracts (BEs) and mycelia extracts (MEs) were prepared separately from each culture and labeled according to salt concentration (i.e. BE0, BE1, ME0, ME1 etc.), then purified as described in Materials and Methods. The strategy is outlined in Figure 1A. Peptides were the main components of MEs, as shown by TLC and stains such as iodide (Figure 1B) or ninhydrin (Figure 1C).

**Figure 2:**
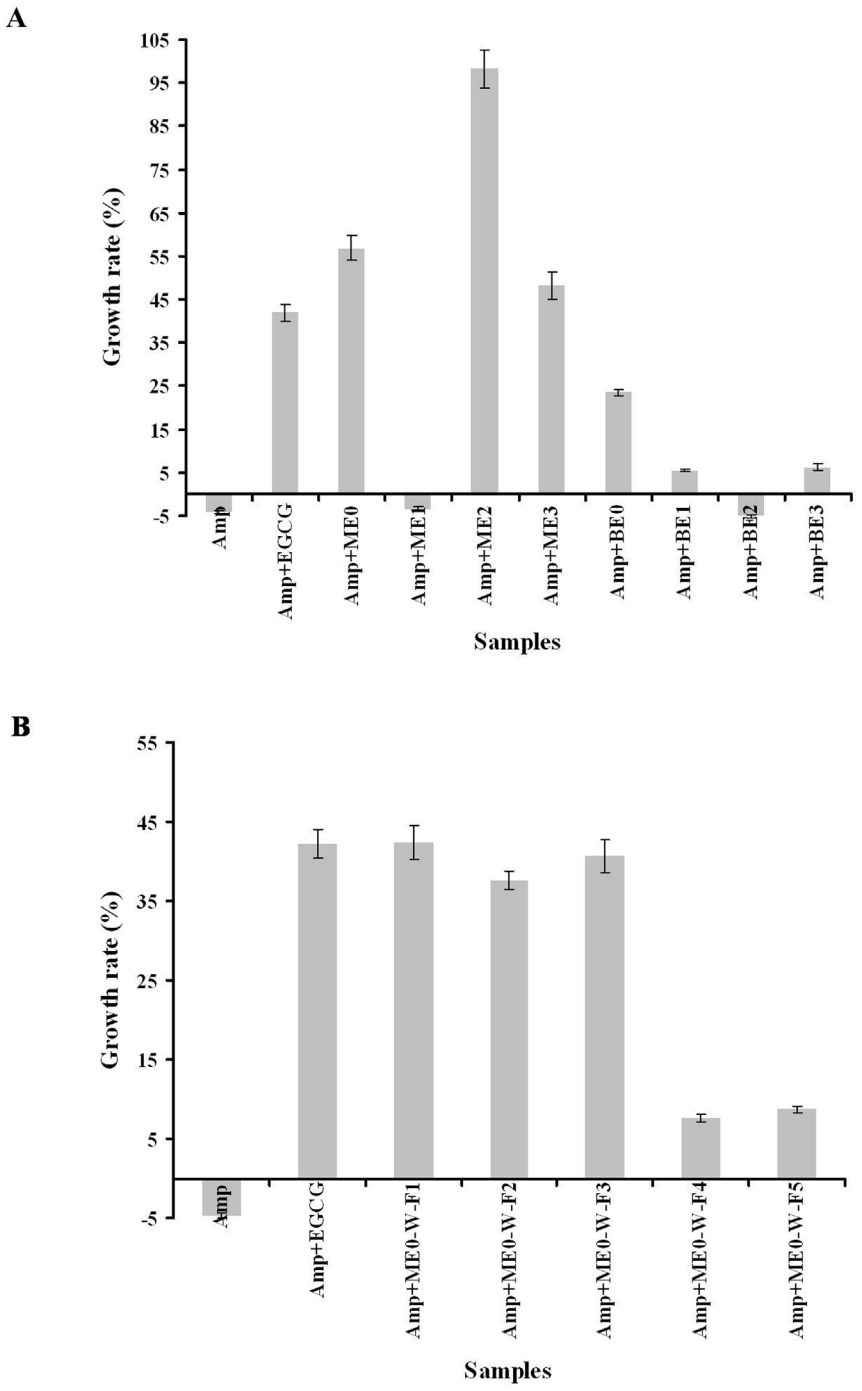
Screening of bioactive fractions from *Ph. occulta* secondary metabolite extracts using an *E. coli* cell model. A: Inhibitory effect on Aβ42 aggregation of *Ph. occulta* secondary metabolite extracts. ME: mycelia extracts; BE: broth extracts; 0, 1, 2 and 3 refer to molar salt concentrations in the cultures. B: Inhibitory effect on Aβ42 aggregation of ME0 fractions. ME0-W-F1 to ME0-W-F5 are water soluble fractions separated by column chromatography using Diaion-20 resin and a water/methanol mobile phase. Fraction concentrations were 200 µg/ml in each case; EGCG was used as positive control (100 µg/ml). Values represent mean of means ± SD of four separate experiments, each performed in triplicate.

The effect of *Ph. occulta* secondary metabolites on the aggregation of Aβ42 was evaluated using an *E. coli* cell model. The fusion protein, ssTorA-Aβ42-Bla, was expressed in *E. coli*. In the presence of samples with Aβ42 aggregation inbititory effect, ssTorA-Aβ42-Bla can be transported into the extracellular space and degrade Amp. Thus, *E. coli* growth is proportional to the inhibitory effect of samples on Aβ42 aggregation [25]. In most cases, growth rates were higher in the presence of MEs than in the presence of BEs. This indicated an inhibitory effect of MEs on the aggregation of Aβ42 in *E. coli*. Relative growth rates of *E. coli* cells with ME0, ME2 and ME3 were 57%, 98% and 48%, respectively, showing that all were at least as effective as the positive control, EGCG, which gave a relative growth rate of 42% (Figure 2A). The *E. coli* growth rate in the presence of ME1 was the same as that of the negative control (no additive), suggesting it had no effect on Aβ42 aggregation.

ME0 was selected for further study and was purified by column chromatography using a Diaion-20 resin with a water/methanol mobile phase. Fractions ME0-W-F1 to ME0-W-F5 were collected and re-tested in the *E. coli* assay: ME0-W-F1, ME0-W-F2 and ME0-W-F3 gave growth rates of 42.31%, 37.60% and 40.68%, respectively, similar to that of EGCG (42%), but ME0-W-F4 and ME0-W-F5 were less effective (Figure 2B). ME0-W-F1, which eluted with 100% H_2_O, consisted largely of water-soluble peptides, and its proportion in ME0 was the highest (75% of the total mass). So, ME0-W-F1 was selected for further research.

### Effect of ME0-W-F1 on Aβ42-induced cytotoxicity in SH-SY5Y cells

**Figure 3:**
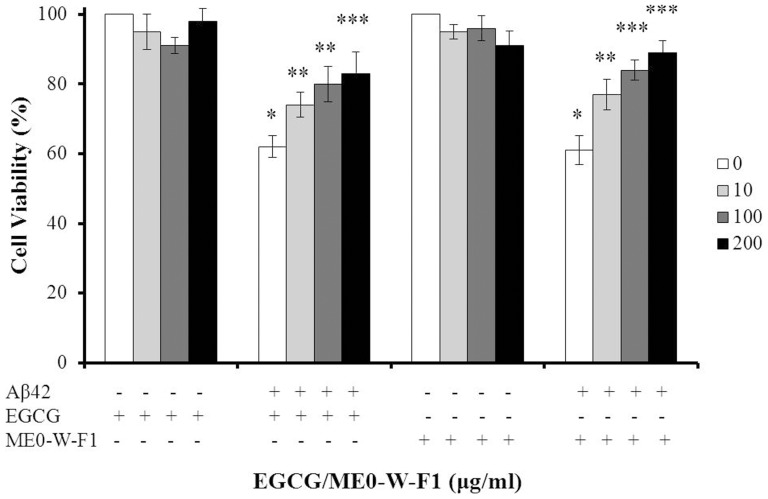
Protective effect of ME0-W-F1 in SH-SY5Y cells against cytotoxicity induced by aggregation of Aβ42 (10 µM), as shown by MTT analysis. Four concentrations (µg/ml) were used, with EGCG as positive control. Values represent mean of means ± SD of four separate experiments, each performed in triplicate (i.e. n = 12). The data were evaluated by two-way analysis of variance (ANOVA) followed by a post hoc test. *, **, ***, statistically significant from each other, *p*<0.05. The treatments with EGCG or ME0-W-F1 were not significant.

An MTT assay in the neuronal cell line SH-SY5Y was employed to explore the cytoprotective activity of ME0-W-F1. We showed that ME0-W-F1 did not affect the viability of SH-SY5Y cells, even at concentrations up to 200 µg/mL (Figure 3). In contrast, exposure to freshly prepared Aβ42 for 48 h was cytotoxic, producing a sharp decrease in SH-SY5Y viability, down to about 62% of control values. When ME0-W-F1 was added, however, the toxic effect of Aβ42 was significantly reduced in a dose-dependent manner, with cell viability of 77%, 84% and 89% at 10 µg/mL, 100 µg/mL and 200 µg/mL, respectively (Figure 3). Thus, ME0-W-F1 can reduce the cytotoxicity of Aβ42 significantly *in vitro*, in a similar fashion to the positive control, EGCG.

### ME0-W-F1 reduces aggregation of fluorescently tagged Aβ42 in HEK293 cells

**Figure 4:**
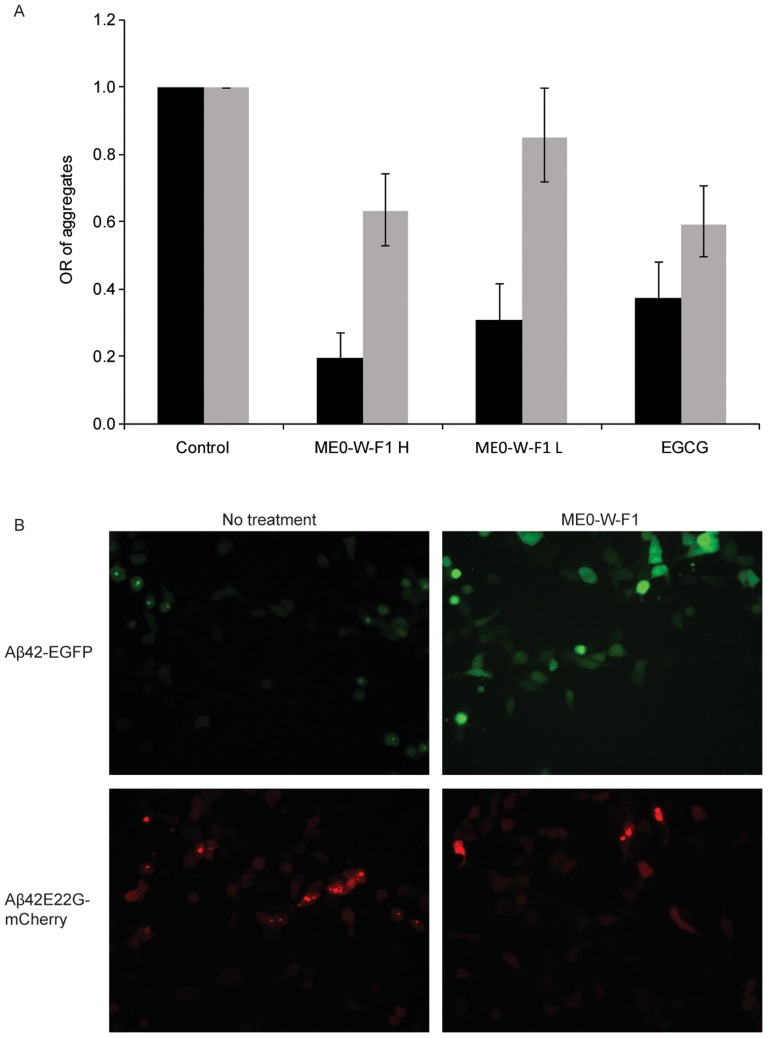
ME0-W-F1 reduces aggregation of Aβ42-EGFP and the early onset familial mutation Aβ42E22G-mCherry). (A, B) HEK293 transiently transfected with pcDNA3-Aβ42–EGFP (grey bars) and the Aβ42E22G-mCherry construct pATNRW20 (black bars) and treated with ME0-W-F1 at 17.5 µg/ml (H) and 1.75 µg/ml (L), a positive control (10 µM EGCG) and a negative control (DMSO only). For each construct and treatment, three fields of approximately 200 cells (i.e. n = 200 for each field) were counted for aggregates and odds ratios calculated. Error bars indicate 95% confidence interval for the odds ratio. Treatments of the Aβ42E22G-mCherry transfections with ME0-W-F1 (H and L) and EGCG were all statistically significant with a probability of *P*<0.0001. Treatments of the Aβ42-EGFP transient transfections with ME0-W-Fl (H) and EGCG were statistically significant with a probability of *P*<0.0001. The treatment with ME0-W-F1 (L) was not significant.

We have previously used Aβ42 aggregation in human cells as a screening tool to identify small molecules with anti-aggregation activity [27]. The effect of ME0-W-F1 was therefore tested in HEK293 cells transiently transfected with genes encoding either Aβ42-EGFP or Aβ42E22G-mCherry; the latter is a mutant form of Aβ42 associated with early onset AD. Both fluorescently tagged forms of Aβ42 aggregated in the human cell line (Figure 4; Figure S1). When ME0-W-F1 was added to cultures 3 h after transfection, however, the number of cells containing aggregates was reduced in an apparently dose-dependent manner. Therefore, ME0-W-F1 contains active components that can suppress aggregation of fluorescently tagged forms of Aβ42 in human cells, similar to the findings in bacteria.

### Inhibitory effect of ME0-W-F1 on Aβ42 aggregation

**Figure 5:**
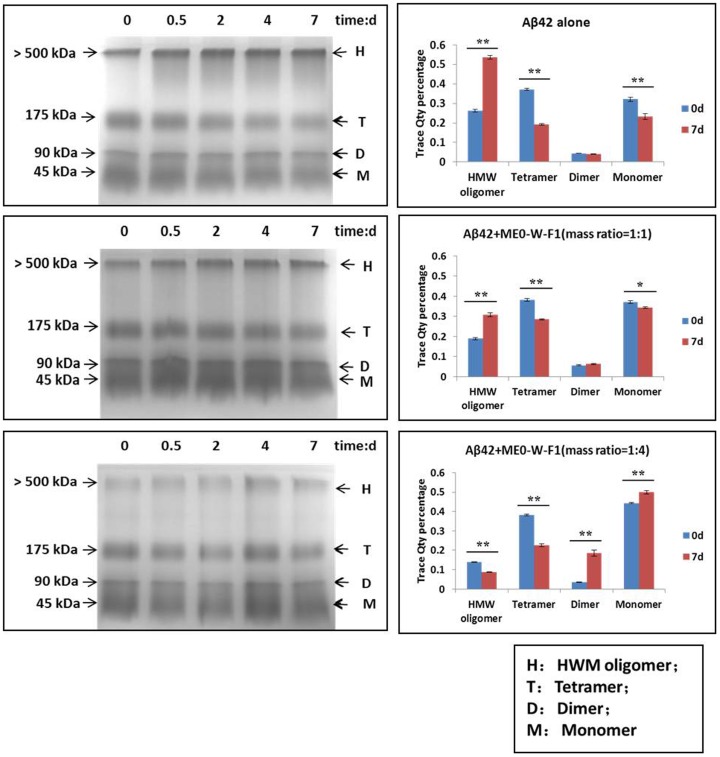
Effect of ME0-W-F1 on aggregation of Aβ42 analysed by SDS-PAGE and quantitative analysis with Quantitative One. Low (Aβ42: ME0-W-F1 = 1∶1) and high (Aβ42: ME0-W-F1 = 1∶4) concentrations of ME0-W-F1 were used. Values represent mean ± SD of three replicates. The data were evaluated by *t*-test, * *p*<0.05, ** *p*<0.01.

SDS-PAGE was used to investigate the effect of ME0-W-F1 on Aβ42 aggregation *in vitro*. When Aβ42 was incubated at 37°C for 7 d in the absence of ME0-W-F1, four main forms of Aβ42 were visible on gels, i.e. monomer, dimer, tetramer and high weight molecular (HMW) oligomers, with the latter comprising about 50% of the material (Figure 5). However, the proportion of Aβ42 forming HMW oligomers was reduced to 32% and 7% after a 7 d incubation under the same conditions in the presence of low and high concentrations of ME0-W-F1, respectively (Figure 5; Figure S2 & S3). There was a corresponding, dose-dependent increase in the amount of Aβ42 monomer when ME0-W-F1 was present, suggesting that the water soluble fraction has an inhibitory effect on Aβ42 oligomerisation.

**Figure 6:**
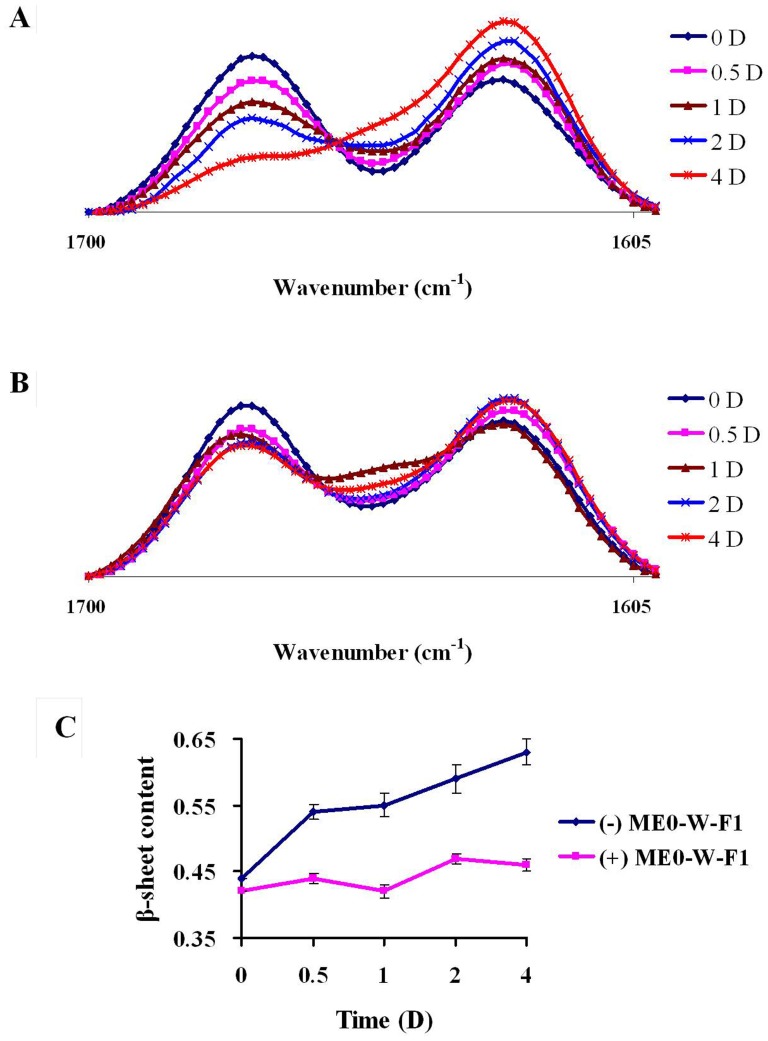
Inhibitory effects of ME0-W-F1 on the transformation of secondary structure of Aβ42 by FT-IR. A: Aβ42 alone; B: Aβ42 with ME0-W-F1; C: change in β-sheet content during incubation with (+) or without (−) ME0-W-F1. Time: 0, 0.5, 1, 2 and 4 days. Values represent mean ± SD of three separate experiments.

The formation of Aβ42 aggregates is characterised by a shift in conformation of the protein secondary structure from α-helix to β-sheet [30]. FT-IR spectroscopy allows this structural transition to be observed in the amide I band, 1600–1700 cm^−1^, in which bands at ∼1670 cm^−1^ and ∼1627 cm^−1^ are characteristic of α-helix and β-sheet, respectively [31], [32]. For Aβ42 alone, there is a progressive shift from α-helix to β-sheets over a 4 d period (Figure 6A). However, this transition is markedly reduced in the presence of ME0-W-F1 at 1 mg/mL (Figure 6B), with the proportion of β-sheet reducing from about 44% to 63% (Figure 6C). These data demonstrate that ME0-W-F1 can disrupt the transformation of α-helix to β-sheet associated with inhibition of the oligomerisation and aggregation of Aβ42.

## Discussion

Aggregation of Aβ into plaques is a hallmark pathogenic feature of dementia and therefore is a primary target for amelioration of the disease [33]. Numerous chemical ligands have been developed as Aβ aggregation inhibitors in recent years including EGCG [34], curcumin [35], scyllo-inositol [36] and LPFFD [37], but very few have progressed to clinical trials. In light of this disappointing situation, it is appropriate to search for alternative Aβ aggregation inhibitors among natural products. Some herb and fungal extracts have remarkable anti-AD activities *in vivo* and *in vitro* due to inhibition of Aβ aggregation [12]–[20]. Such studies justify further research on natural products, which could identify candidate lead compounds for AD treatment.

Evidence is accumulating that fungi are more likely to produce novel chemicals when they live in extreme environments [38]. Mangrove endophytic mycelium as a source of new microorganisms with potential pharmaceutical value has been intensively researched in recent years [39], [40]. In this paper, the inhibitory effects of secondary metabolites of *Ph. occulta*, a new endophytic fungus isolated from roots of the mangrove *Po. pinnata* (L.) Pierre, on the aggregation of Aβ42 *in vitro* and in cells were reported. Although its growth rate declined as the concentration of NaCl increased, *Ph. occulta* could be fermented in the presence of salt at various concentrations. This confirms *Ph. occulta* as a salt tolerant endophytic fungus.

The bioactivity of secondary metabolites from *Ph. occulta* is affected by the salt conditions during its growth. Thus, ME1, extracted from fungi grown at 1 M NaCl, has no clear growth-promoting, and hence anti-aggregation, effect in an *E. coli* model of Aβ42 aggregation. This might be because *Ph. occulta* grows naturally in sea water, which contains about 0.75 M NaCl, and is less stressed at 1 M NaCl than lower or higher salt concentrations. In contrast, MEs from *Ph. occulta* grown at 0 M, 2 M and 3 M NaCl exhibited strong growth-promoting effects in the *E. coli* model, suggesting that certain secondary metabolites produced under such salt stress conditions have anti-aggregation activity. The water-soluble peptides in the selected bioactive fraction, ME0-W-F1, are candidates for such secondary metabolites. Because growth of *Ph. occulta* is very slow under high salt (2 M and 3 M NaCl) conditions, it was not possible to obtain sufficient quantities of material to test fractions such as ME2, which had strong bioactivity (Figure 2). Therefore, our analysis was limited to ME0 and related fractions. The BEs had no inhibitory effects on the aggregation of Aβ42.

ME0-W-F1 is active in human cells, as well as the *E. coli* model, reducing the cytotoxicity of Aβ42 in the SH-SY5Y cell line. Aβ oligomerisation and fibril formation are toxic to neurons, and these processes mediate Aβ toxicity mainly through interaction with other factors, e.g. Tau, in AD [5]. This suggests that ME0-W-F1 antagonises the oligomerisation and aggregation of Aβ42. An effect of ME0-W-F1 on intracellular Aβ42 aggregation was demonstrated in a HEK293 cell line, in which the water-soluble fraction reduced aggregation of both Aβ42 expressed as a fusion protein with EGFP and also an early onset form, Aβ42E22G, fused to mCherry; the fluorescent fusion partners allowed visualisation of aggregates within cells.

During the aggregation of Aβ *in vivo*, it is suggested that native Aβ peptides undergo conformational changes to form misfolded intermediates and various aggregated structures rich in β-sheet [41]. The transformation from α-helix to β-sheet is thought to be the rate-limiting step in the formation of soluble Aβ intermediates and oligomers, which are the most toxic Aβ species and are typically unstable, undergoing further aggregation to form higher-order oligomers and fibrillar deposits [7], [42]. *In vitro*, ME0-W-F1 inhibits this structural transition from α-helix to β-sheet, as shown by both SDS-PAGE and FT-IR spectroscopy. Thus, SDS-PAGE demonstrated that the formation of tetramers and HMW oligomers of Aβ42 was disrupted in the presence of ME0-W-F1 in a dose- and time-dependent manner. Similarly, FT-IR spectroscopy showed that the shift from α-helix to β-sheet as Aβ42 aggregated was markedly reduced when ME0-W-F1 was present. These results suggest that ME0-W-F1 inhibits the oligomerisation and aggregation of Aβ42 through blocking the transformation of secondary structure and preventing subunit assembly.

Since ME0-W-F1 prevented or reduced the aggregation of intracellular Aβ42 fusion proteins in both bacteria and human cells, and since *in vitro* studies suggest an interaction with Aβ42 species, it seems likely that the active components of the water-soluble fraction must gain access to the intracellular space. The most likely mechanism of action is that these components interfere with Aβ42 aggregation within cells, as occurs *in vitro*, but we cannot rule out, for example, a stimulatory effect on molecular chaperone surveillance systems. The nature of the active components of ME0-W-F1 and the molecular mechanism of their action are currently under investigation.

In summary, water-soluble secondary metabolites from *Ph. occulta* exhibited inhibitory effects on the oligomerisation and aggregation of Aβ42 in cells and *in vitro*. Therefore, ME0-W-F1 and *Ph. occulta* are novel natural materials worthy of further investigation as potential therapeutic agents for AD.

## Supporting Information

Figure S1 **Confocal microscopy of Aβ42-EGFP and Aβ42E22G-mCherry aggregates in HEK293 cells.** A: Aβ42-EGFP expression, showing DAPI staining of nuclei (blue), EGFP fluorescence (green) and a transmitted light image, together with a merged image. The main aggregate is visible as a bright fluorescent spot located adjacent to the nucleus in one cell. Non-aggregated Aβ42-EGFP can be seen as a less intense green fluorescence in the cytoplasm of this and other transfected cells. B: Aβ42E22G-mCherry expression, showing DAPI staining of nuclei (blue), mCherry fluorescence (red) and a transmitted light image, together with a merged image. Large aggregates are visible as bright fluorescent spots distributed around and within the nuclei of several cells. Non-aggregated Aβ42E22G-mCherry can be seen as a less intense red fluorescence in the cytoplasm of these cells.
(TIF)

Figure S2 **Effect of ME0-W-F1 on aggregation of Aβ42 analysed by SDS-PAGE.** Low (Aβ42: ME0-W-F1 = 1∶1) concentration of ME0-W-F1 was used.
(TIF)

Figure S3 **Effect of ME0-W-F1 on aggregation of Aβ42 analysed by SDS-PAGE.** High (Aβ42: ME0-W-F1 = 1∶4) concentration of ME0-W-F1 was used.
(TIF)
