# Correction: Effects of Secondary Metabolite Extract from *Phomopsis occulta* on β-Amyloid Aggregation

**DOI:** 10.1371/journal.pone.0117542

**Published:** 2015-01-28

**Authors:** The PLOS ONE Staff

## Notice of Republication

This article was republished on December 19, 2014, to remove confidential information. The publisher apologizes for the error, which should have been corrected during the review process.
