# Supplementary figures and images for: Effects of Secondary Metabolite Extract from Phomopsis occulta on β-Amyloid Aggregation
Source: PLoS One. 2014 Oct 2;9(10):e109438. doi: 10.1371/journal.pone.0109438 (PMC4183696; doi:10.1371/journal.pone.0109438)

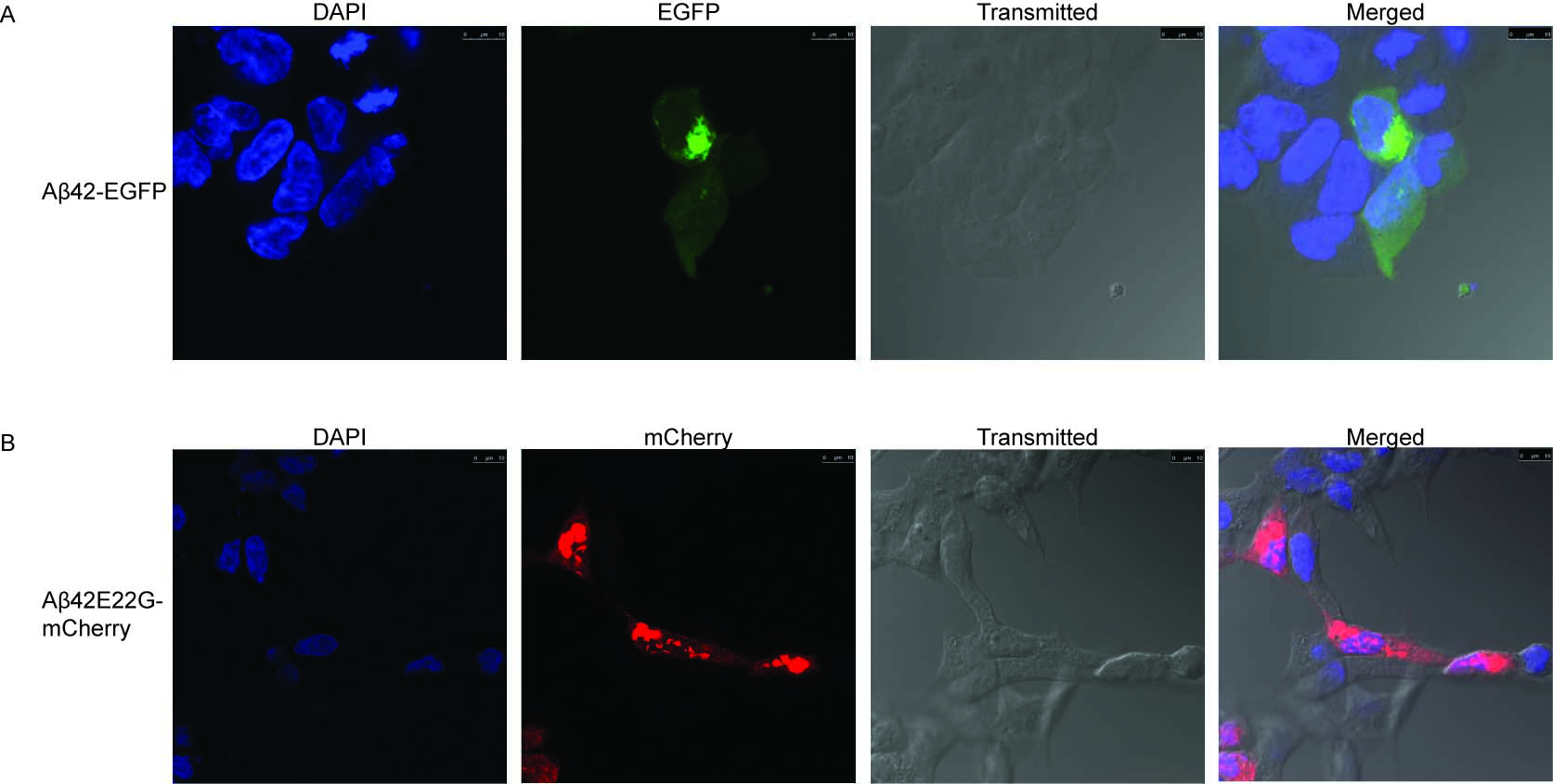

Supplement: Figure S1 — Confocal microscopy of Aβ42-EGFP and Aβ42E22G-mCherry aggregates in HEK293 cells. A: Aβ42-EGFP expression, showing DAPI staining of nuclei (blue), EGFP fluorescence (green) and a transmitted light image, together with a merged image. The main aggregate is visible as a bright fluorescent spot located adjacent to the nucleus in one cell. Non-aggregated Aβ42-EGFP can be seen as a less intense green fluorescence in the cytoplasm of this and other transfected cells. B: Aβ42E22G-mCherry expression, showing DAPI staining of nuclei (blue), mCherry fluorescence (red) and a transmitted light image, together with a merged image. Large aggregates are visible as bright fluorescent spots distributed around and within the nuclei of several cells. Non-aggregated Aβ42E22G-mCherry can be seen as a less intense red fluorescence in the cytoplasm of these cells. (TIF) [file pone.0109438.s001.tif]

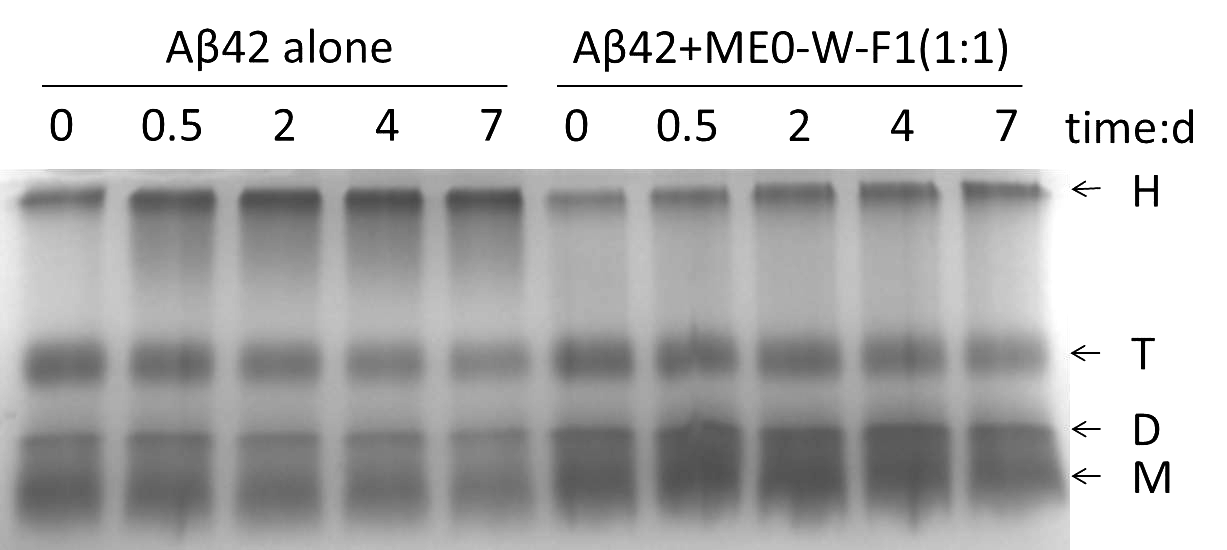

Supplement: Figure S2 — Effect of ME0-W-F1 on aggregation of Aβ42 analysed by SDS-PAGE. Low (Aβ42: ME0-W-F1 = 1∶1) concentration of ME0-W-F1 was used. (TIF) [file pone.0109438.s002.tif]

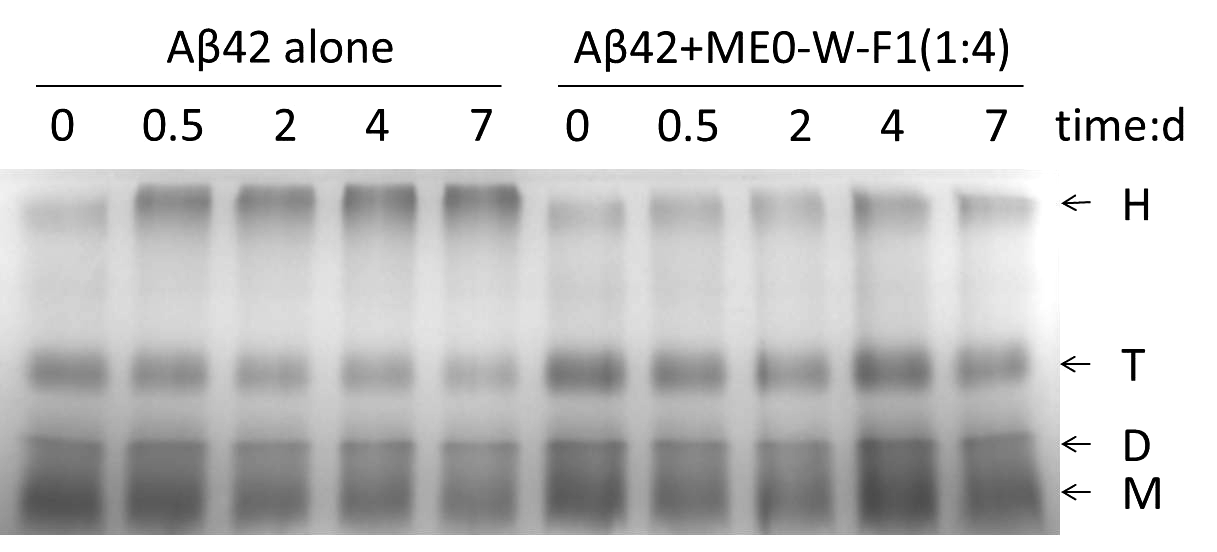

Supplement: Figure S3 — Effect of ME0-W-F1 on aggregation of Aβ42 analysed by SDS-PAGE. High (Aβ42: ME0-W-F1 = 1∶4) concentration of ME0-W-F1 was used. (TIF) [file pone.0109438.s003.tif]
